# Bone marrow non-mesenchymal mononuclear cells induce functional differentiation of neuroblastoma cells

**DOI:** 10.1186/2162-3619-2-9

**Published:** 2013-04-03

**Authors:** Chareerut Phruksaniyom, Permphan Dharmasaroja, Surapol Issaragrisil

**Affiliations:** 1 Department of Anatomy, Faculty of Science, Mahidol University, Rama VI Road, Ratchathewi, Bangkok, 10400, Thailand; 2 Division of Hematology, Department of Medicine, Faculty of Medicine Siriraj Hospital, Mahidol University, Bangkok, 10700, Thailand

**Keywords:** Bone-marrow mononuclear cells, Neuroblastoma cells, Tyrosine hydroxylase, Trophic factors, Neuronal differentiation

## Abstract

Less is known about the non-mesenchymal mononuclear cell fraction of human bone marrow on functional adaptation of neuroblastoma cells. Using immunocytochemistry, we showed that bone-marrow mononuclear cell (BMMC)-conditioned medium can induce tyrosine hydroxylase expression in neuroblastoma cells, which is similar to the effect of retinoic acid. Using quantitative RT-PCR, we showed that NGF, CNTF, and BDNF mRNAs were detected in unfractionated BMMC populations from all human donors at different expression levels. Our results suggest that cells of the non-mesenchymal mononuclear cell fraction can induce functional adaptation of neuroblastoma cells, probably via their secreted trophic factors.

## To the editor

Many studies investigating the possible therapeutic role of bone marrow-derived stem cells (BMDCs) used a specific subpopulation: the bone marrow mesenchymal stromal cells (MSCs) obtained after several weeks in cultures [1], or the mononuclear fraction (BMMC; bone marrow mononuclear cells) obtained immediately after aspiration. Cells of the hematopoietic stem cell fraction, when transplanted into lesions of a developing spinal cord in a chicken embryo, can differentiate into neurons [2]. The capacity of BMMCs to generate neural cells is poorly characterized. Several studies indicate an overlap in the molecular programs for hematopoiesis and neuropoiesis in mice [3,4]. Primary CD34+ human hematopoietic stem cells (HSCs) have been shown to express mRNA for a number of proteins that are used by neurons [5].

Evidence has indicated that human SH-SY5Y neuroblastoma cells changed into neuron-like phenotypes with reduced proliferation by *all-trans* retinoic acid (ATRA) [6], and treatment with RA increased protein expression of tyrosine hydroxylase (TH) in neuroblastoma cells [7]. Using a co-culture method, human MSCs promoted the survival and neuritogenesis of neuroblastoma cells, similar to that of ATRA [8]. Less is known about the mononuclear cell fraction of human bone marrow on functional adaptation of neuroblastoma cells.

**Figure 1:**
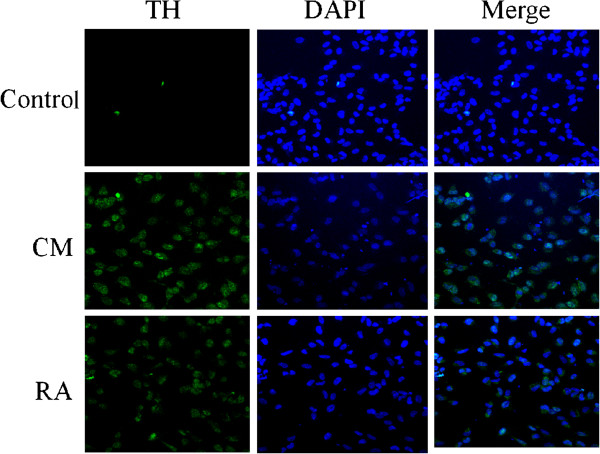
**Immunostaining micrographs using a confocal microscope demonstrate localization of the tyrosine hydroxylase (TH).** Cells were stained with polyclonal antibody against TH and immunostained with Alexa 488-conjugated secondary antibody (green) and nuclei were stained with DAPI (blue). CM, BMMC-conditioned medium; RA, retinoic acid.

We hypothesize that cells of the BMMC fraction can induce functional adaptation of neuroblastoma cells, probably via their secreted trophic factors. First, we evaluated the effect of cells of the human BMMC fraction on the expression of TH protein in neuroblastoma cells by culturing SH-SY5Y cells in BMMC-conditioned medium. Human bone marrow samples were aspirated from healthy donors after obtaining informed consent and ethical approval by the Siriraj Ethics Committee of Siriraj Hospital. After isolation, the mononuclear cells were plated at a concentration of 1.5 × 10^5^ cells/ml onto flasks containing low glucose-DMEM supplemented with 10% fetal bovine serum (FBS). After incubation for 72 h, non-adherent cells were collected for culture in MEM/F12 medium supplemented with 10% FBS. After an additional 24 h, non-adherent cells were collected again and cultured in the medium as described above. After 24 h, non-adherent cell-conditioned medium was collected for further experiment. SH-SY5Y cells were then seeded onto plates containing BMMC-conditioned medium at an initial density of 1 × 10^4^ cells/ml, and cultures were maintained for 7 days prior to analysis of TH expression using immunocytochemistry. As a positive control, cells were treated with 10 μM ATRA for 5 days to induce neuronal differentiation. With estimated concentration of 35 ± 2 × 10^4^ cells/ml for staining, the results showed that BMMC-conditioned medium can induce TH protein expression in neuroblastoma cells (Figure 1C), which is similar to the effect of ATRA (Figure 1B). Untreated cells did not express TH or expressed at very low levels (Figure 1A).

**Figure 2:**
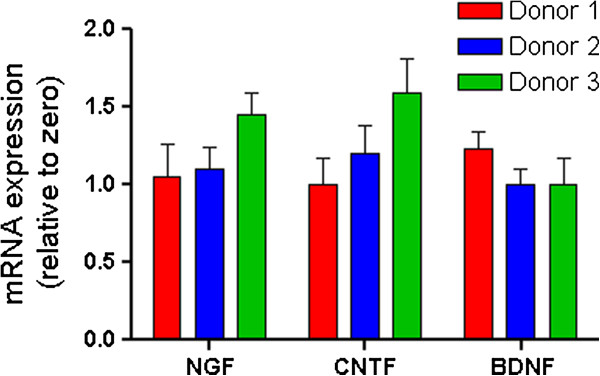
**Quantitative expression of NGF, CNTF, and BDNF mRNAs in cells of the BMMC fraction obtained from three healthy donors.** 20 ng cDNA was used as PCR template. Real-time PCR mixture was prepared with KAPA SYBR® FAST qPCR master mix. β-actin was used as the reference gene. Each bar represents mean ± SD from triplicate of each sample. The expression levels were scaled relative to the lowest unscaled expression level for the same gene as the sample of interest.

Further, we evaluated whether cells of the BMMC fraction expressed any trophic factors that could contribute to biochemical adaptation of neuroblastoma cells. Monocytes in the human bone marrow have been shown to produce nerve growth factor (NGF), which plays an important role in neuronal plasticity, maturation, and survival [9]. Human monocytes, T cells, and B cells can secrete brain-derived neurotrophic factor (BDNF), a member of the neurotrophin family that regulates the differentiation and survival of various neuronal populations [10]. Ciliary neurotrophic factor (CNTF), another factor involved in neurogenesis, is also expressed in monocytes, myeloid cells, lymphoblasts, T cells and B cells [11]. Here, we evaluated the mRNA expression of NGF, BDNF, and CNTF in BMMCs using quantitative RT-PCR. The sequences of the sense and antisense primers are as follows: NGF: TAAAAAGCGGCGACTCCGTT and ATTCGCCCCTGTGGAAGATG; CNTF: ACCAGCAGGTGCATTTTACC and GAAACGAAGGTCATGGATGG; BDNF: ACTCTGGAGAGCGTGAATGG and ATCCAACAGCTCTTCTATCACG; β-actin: CATGTACGTTGCTATCCAGGC and CTCCTTAATGTCACGCACGAT. The results showed that NGF, CNTF, and BDNF mRNAs were detected in unfractionated BMMC populations from all donors at different expression levels (Figure 2). Their expression levels were rather low, suggesting that not all but only some populations of the cells expressed these trophic factors.

Expression of TH, the enzyme involved in the first step of the biosynthesis pathway of dopamine and noradrenaline, in SH-SY5Y cells by BMMC-conditioned media suggests the functional differentiation of the cells. ATRA can also induce responsiveness to BDNF in SH-SY5Y cells [12]. Our results imply that expression and secretion of BDNF from cells in the mononuclear fraction may explain the similar effects of RA and BMMC-conditioned media. BMDCs constitutively synthesize and secrete NGF, BDNF, and CNTF. Multiple cell types, however, are present in the BMMC fraction. Primary CD34+ human HSCs express mRNA for a number of proteins, including receptors for trophic factors and other mediators involved in the development of neurons [5]. Most of the CD34+ cells are progenitors for myeloid and lymphoid lineages, which express some trophic factors, such as CNTF [11], that were also observed in our quantitative RT-PCR results. Further studies are required to quantify the contribution of trophic factors to BMMC-induced effects on functional adaptation of neuroblastoma cells, which could have clinical relevance in treatment of neuroblastoma.

## Competing interests

The authors declare that they have no competing interests.

## Authors’ contributions

PD was involved in the design and execution of the experiments, performed data and statistical analyzes, wrote the manuscript and contributed to overall experiment design. CP conducted immunocytochemistry and quantitative RT-PCR. SI provided bone marrow samples and submitted the project for ethical approval. All authors have read and approved the final manuscript.
